# Lateral cervical thymic cyst in a child: a case report

**DOI:** 10.1186/s13104-018-3208-x

**Published:** 2018-01-30

**Authors:** F. E. Hazmiri, F. Nachite, D. Skandour, A. Raji, N. Cherif Idrissi El ganouni, H. Rais

**Affiliations:** 1 Department of Pathology, FMFM-UCAM-CHU Mohammed VI-50 Assif, 40000 Marrakech, Morocco; 2 Department of E.N.T, FMFM-UCAM-CHU Mohammed VI-50 Assif, Arrazi Hospital, 40000 Marrakech, Morocco; 3 Department of Radiology, FMFM-UCAM-CHU Mohammed VI-50 Assif, Arrazi Hospital, 40000 Marrakech, Morocco

**Keywords:** Thymic cyst, Ectopic tissue, Thymus gland, Hassall’s corpuscles

## Abstract

**Background:**

Cervical thymic cysts are uncommon lesions, rarely considered in the differential diagnosis of neck cysts in children.

**Case presentation:**

We report a rare case of multiloculated thymic cyst in an 8-year-old boy on the right side of the neck. Perioperative diagnosis was a cystic hygroma. Macroscopic examination showed a cystic mass measuring 6.5 cm in total length. Histopathology of the excised specimen revealed thymic tissue with prominent Hassall’s corpuscles associated with multiloculated cyst. The cyst wall is bordered by a flattened or multilayered epithelium, often abraded.

**Conclusion:**

This case is presented here for its rarity and should be included in the differential diagnosis of neck masses in children. So, it’s a lesion to be well aware of, particularly by pathologists.

## Background

The cervical thymic cyst is an uncommon and poorly known malformation of embryological origin [1]. Heise and al. reported cervical thymic cysts to be 0.3% of all congenital cervical cysts in children [2]. Due to its rarity, it is not generally considered in the differential diagnosis of pediatric cystic neck masses. Thereby, it is often confused with the branchial cleft cyst, much more frequent, the cystic lymphangioma or the thyroglossal duct cyst [3], and it is invariably diagnosed on histopathology [4, 5]. We report a new observation of cervical thymic cyst in an 8-year-old boy, erroneously diagnosed as a cystic hygroma preoperatively but confirmed by pathological examination.

## Case presentation

An 8-year-old boy presented to ENT consultation for a right laterocervical swelling, discovered by his parents 2 years ago. The swelling was painless without altering its volume and it was not associated with signs of cervical compression.

**Fig. 1:**
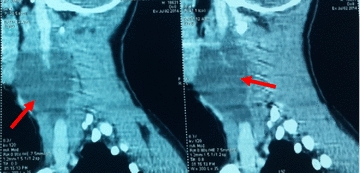
Cervical CT with parasagittal reconstruction showing a thin walled, prevertebral and laterocervical cystic mass, with intimate vascular contacts (red arrow)

**Fig. 2:**
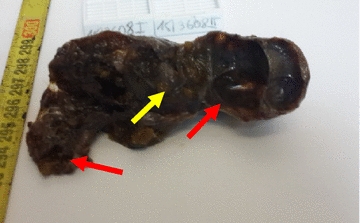
Oblong mass with a multilocular cystic aspect (red arrows) in cut section and multiple brownish parietal nodules (yellow arrow)

**Fig. 3:**
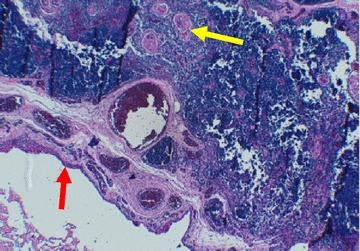
Cystic wall bordered by a cubic or multilayered epithelium (red arrow) and comprising a lymphoid tissue with Hassall’s corpuscles (yellow arrow) (HE X 10)

**Fig. 4:**
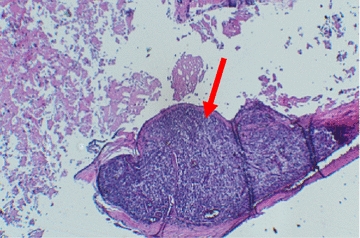
Parathyroid parenchyma found in the cystic wall (red arrow) (HE X 10)

Physical examination revealed a mass of about 5 cm well delineated, soft to tense cystic in feel, painless, with no inflammatory signs or palpable adenopathy. The complete and general ENT examination did not show abnormalities. Ultrasound showed a multiloculated cystic mass of 5.4 cm, with thick contents. This aspect evoked a cystic hygroma or a bronchogenic cyst. Cervical CT revealed the same ultrasound signs of the mass. This mass had intimate vascular contacts and was accompanied by bilateral infracentimetric jugulo-carotid lymphadenopathies but without invasion of the vicinity. The mass walls enhanced discreetly to the injection of contrast agent (Fig. 1). At this stage, the diagnosis of cystic lymphangioma was advanced. In perioperative, the mass adhered deeply to the sternocleidomastoid and had an intimate relationship with the carotid sheath. Macroscopic examination showed an oblong cystic mass measuring 6.5 cm in total length. In section, the mass unveiled a multilocular appearance. The cystic cavities were between 0.5 and 3.5 cm in diameter and contained slightly thick and light brown material. Walls were thick in places and often presented tiny brownish nodules (Fig. 2). Histopathological examination showed a cyst wall bordered by a flattened or multilayered epithelium, often abraded and surmounting islands of thymic tissue (Fig. 3). The latter contained cholesterol granulomas and Hassall’s corpuscles with cystic degeneration in places. On one of the histological sections, the parathyroid parenchyma was embedded in the cyst wall (Fig. 4). Thus, the diagnosis of cervical thymic cyst was made postoperatively. The short-term evolution was favorable without incident or recidivism noted.

## Discussion and conclusions

Embryologically, the thymus, a paired organ, arises from the third and fourth pharyngeal pouches. During the sixth week of intrauterine life, the right and left thymic flaps proliferate and then merge to form the thymopharyngeal duct. The latter extends from the mandibular angle to the mediastinum and guides thymus descent towards the mediastinum. The upper part of this canal regresses thereafter and any failure of its closure would lead to the formation of the congenital unilocular cervical thymic cyst. On the other hand, the degeneration of Hassall’s corpuscles within rests of ectopic thymus tissue would be at the origin of the acquired multilocular thymic cyst. Thus, two main theories of cervical thymic cyst pathogenesis are proposed by Speer; firstly the persistence of the thymopharyngeal duct and secondly the degeneration of Hassall’s corpuscles [6–9].

Furthermore, the development of the parathyroid glands is intimately connected with that of thymus. Embryologically, the thymus is derived from the 3rd and 4th pair of parapharyngeal pouches as are the parathyroids, thus, a small amount of parathyroid tissue was reported to be found adjacent to the wall of thymic cyst [9]. However, the two organs are not considered as a unit since they assure different functions once their development is finished.

The cervical thymic cyst is a rare entity with only about 100 cases reported in the literature [3]. The mean age of onset is 7 years [5] with a slight male predominance [8]. Most cases are asymptomatic, making diagnosis difficult [3, 4]. Usually, the lesion is found anterior to sternocleidomastoid [4] along the embryological line of the thymus descent [3]. It occurs on the left side in 60–70% of patients [6]. In 6–13% of cases, the patient may present with stridor, dysphonia or dysphagia [4]. The most important radiological exams are CT scan and MRI. They specify the relation of the cyst to the great vessels of neck, differentiate cervical thymic cysts from branchial cleft cysts and lymphangiomas, then evaluate the mediastinal extension of the lesion [5, 6, 8].

Treatment is based on surgical excision [1, 5, 8]. However, it is critical that the existence of a mediastinal thymus must be confirmed with MRI or FNAC prior to surgery because thymectomy during early childhood can impair immune status later in life [4, 8, 10]. Based on Rahmati’s series [11] where patients with asymptomatic cysts incidently discovered were operated on in order to confirm the histological nature of the lesion, we think it’s prudent to do the same on all incipient/small cervical cysts which are sure to be asymptomatic. Pathological examination shows at the macroscopic level a cyst often elongated uni or multilocular measuring between 1 and 15 cm in diameter [6], with clear to brownish serous contents [8]. Histopathological examination is the only definitive means of diagnosis [4, 5]. The cyst contains ectodermal derivative, e.g. epithelium ranging from squamous to cuboidal and columnar cells. It also contains endodermal derivatives, e.g. thymic and parathyroid tissue. The diagnosis of a cervical thymic cyst depends on the finding of thymic tissue and Hassall’s corpuscles [5]. The pathological differential diagnosis is mainly made with a branchial cleft cyst, lymphadenopathy with cystic degeneration and a dermoid cyst. More rarely, it may include a thyroglossal duct cyst or whether a thyroid or parathyroid cyst [5].

The cervical thymic cyst never undergoes a malignant transformation, unlike the ectopic and non-cystic solid thymic tissue [4–6].

Cervical thymic cysts are rare but should be considered in the differential diagnosis of lateral neck swellings especially in the pediatric population.

Definitive diagnosis depends on histopathological examination. Hence knowledge of this entity to pathologists will prevent its misdiagnosis.

Surgery is the basic treatment after ruling out the possibility of immunological disturbance especially in young children.

Prognosis is excellent.

## Abbreviations

ENT: ear, nose and throat
CT: computed tomography
MRI: magnetic resonance imaging
FNAC: fine needle aspiration cytology

## References

[1] Leloupa P, Malard O, Stalder JF, Barbarot S (2012). Kystes et fistules congénitaux de la face et du cou. Annales de dermatologie et de vénéréologie.

[2] Heise YY, Hsue S, Lin JN (2003). Pathological analysis of congenital cervical cysts in children: 20 years of experience at Chang Gung Memorial Hospital. Chang Gung Med J.

[3] Tandon A, Tandon R, Chandrashekhar M, Das P, Bansal B, Bhatia N. Bhatia. Cervical ectopic thymic cyst: a rare preoperative diagnosis. BMJ Case Rep. 2011 Oct 4; 2011. 10.1136/bcr.05.2011.4250. 10.1136/bcr.05.2011.4250 PMC3189656 22679153

[4] Srivalli M, Qaiyum HA, Srinivas Moorthy PN, Srikanth K (2011). A case report of cervical thymic cyst and review of literature. Indian J Otolaryngol Head Neck Surg.

[5] Iqbal SMd, Garg AK, Dubey A (2005). Cervical thymic cyst—a case report. Indian J Otolaryngol Head Neck Surg..

[6] Jaiswal AA, Garg AK, Ravindranath M, Mohanty MK (2014). Multiloculated cervical thymic cyst—case report with review of literature. Egypt J Ear Nose Throat Allied Sci.

[7] Speer FD (1938). Thymic cysts. Bull N Y Med Coll.

[8] Prabhakar G, Santhosh AN, Manjunath SS, Santosh KV (2013). Cervical thymic cyst: a case report. Indian J Otolaryngol Head Neck Surg.

[9] Jindal A, Sukheeja D (2016). Unilateral cervical thymic cyst in a child: a rare case report. J Sci Soc..

[10] Sturm JJ, Dedhia K, Chi DH (2017). Diagnosis and management of cervical thymic cysts in children. Cureus.

[11] Rahmati M, Corbi P, Gibelin H, Jayle C, Abdou M, Milinkevitch S, Menu P, Kraimps JL (2004). Prise en charge des kystes thymiques. Management of thymic cysts. Ann Chir.

